# Stretch Causes cffDNA and HMGB1-Mediated Inflammation and Cellular Stress in Human Fetal Membranes

**DOI:** 10.3390/ijms25105161

**Published:** 2024-05-09

**Authors:** Justin Gary Padron, Chelsea A. Saito Reis, Po’okela K. Ng, Nainoa D. Norman Ing, Hannah Baker, Kamalei Davis, Courtney Kurashima, Claire E. Kendal-Wright

**Affiliations:** 1 Anatomy, Biochemistry and Physiology, John A. Burns School of Medicine, University of Hawai‘i at Mānoa, Honolulu, HI 96822, USA; jpadron@wayne.edu; 2 Wayne State School of Medicine, Detroit, MI 48201, USA; 3 Natural Science and Mathematics, Chaminade University of Honolulu, Honolulu, HI 96816, USA; csaitoreis@gmail.com (C.A.S.R.); spencerkamakane@gmail.com (P.K.N.); nainoa.normaning@student.chaminade.edu (N.D.N.I.); bakerbakerhannah@gmail.com (H.B.); kamalei.davis@student.chaminade.edu (K.D.); courtney.kurashima@student.chaminade.edu (C.K.); 4 Obstetrics, Gynecology and Women’s Health, John A. Burns School of Medicine, University of Hawai‘i at Mānoa, Honolulu, HI 96826, USA

**Keywords:** danger-associated molecular patterns, human fetal membranes, stretch, rupture of the membranes, cell-free fetal DNA, high-mobility group box-1

## Abstract

Danger-associated molecular patterns (DAMPs) are elevated within the amniotic cavity, and their increases correlate with advancing gestational age, chorioamnionitis, and labor. Although the specific triggers for their release in utero remain unclear, it is thought that they may contribute to the initiation of parturition by influencing cellular stress mechanisms that make the fetal membranes (FMs) more susceptible to rupture. DAMPs induce inflammation in many different tissue types. Indeed, they precipitate the subsequent release of several proinflammatory cytokines that are known to be key for the weakening of FMs. Previously, we have shown that in vitro stretch of human amnion epithelial cells (hAECs) induces a cellular stress response that increases high-mobility group box-1 (HMGB1) secretion. We have also shown that cell-free fetal DNA (cffDNA) induces a cytokine response in FM explants that is fetal sex-specific. Therefore, the aim of this work was to further investigate the link between stretch and the DAMPs HMGB1 and cffDNA in the FM. These data show that stretch increases the level of cffDNA released from hAECs. It also confirms the importance of the sex of the fetus by demonstrating that female cffDNA induced more cellular stress than male fetuses. Our data treating hAECs and human amnion mesenchymal cells with HMGB1 show that it has a differential effect on the ability of the cells of the amnion to upregulate the proinflammatory cytokines and propagate a proinflammatory signal through the FM that may weaken it. Finally, our data show that sulforaphane (SFN), a potent activator of Nrf2, is able to mitigate the proinflammatory effects of stretch by decreasing the levels of HMGB1 release and ROS generation after stretch and modulating the increase of key cytokines after cell stress. HMGB1 and cffDNA are two of the few DAMPs that are known to induce cytokine release and matrix metalloproteinase (MMP) activation in the FMs; thus, these data support the general thesis that they can function as potential central players in the normal mechanisms of FM weakening during the normal distension of this tissue at the end of a normal pregnancy.

## 1. Introduction

The human fetal membrane (FM) is a multilayered tissue that serves as a physical and immunological barrier for the protection of the fetus throughout gestation [1,2]. Although it is crucial for the success of a pregnancy, the sequence of biochemical and biophysical processes that lead to its rupture in normal pregnancy are not fully understood. Composed of an inner amnion, chorion, and outer decidua, the FM must maintain its integrity until rupture to prevent fetal exposure to ascending infection and other cellular stressors including oxidative stress (OS), tissue distension, and danger-associated molecular patterns (DAMPs).

Evidence suggests that DAMPs may play a central role in the initiation of parturition by contributing to the mechanisms that predispose the FMs to rupture. DAMPs are molecules that are released, activated, or secreted in response to tissue injury and by damaged or dying cells [3]. They are well-studied for their roles as proinflammatory effectors and biomarkers of cellular stress [4,5]. DAMPs, such as high-mobility group box-1 (HMGB1) and cell-free fetal DNA (cffDNA), have been shown to trigger FM inflammation by the release of proinflammatory cytokines known to contribute to the initiation of parturition, such as tumor necrosis factor alpha (TNF-α), interleukin 6 (IL-6), and interleukin 8 (IL-8), and are therefore thought to be key players in FM weakening. Intraamniotic elevation of DAMPs are associated with increased gestational age and labor [6,7,8], although the triggers that initiate their release in utero have not been well established.

HMGB1 is one of the few DAMPs that have been studied in FMs. HMGB1 has also been shown to be released by human amnion epithelial cells (hAECs) as a result of mechanical stretch and OS, and this can drive inflammation throughout the different stages of pregnancy [9,10,11]. cffDNA is a less studied DAMP in the FM, but it is also found in high concentrations in the amniotic fluid [12]. cffDNA also has utility as a biomarker in maternal serum for the detection of various pregnancy complications such as preeclampsia and preterm birth. Similar to HMGB1, cffDNA can elicit the secretion of proinflammatory cytokines and Matrix Metalloproteinases (MMPs) from human FMs [13,14]. Indeed, the specific responses from this DAMP are known to be dependent on the size of the cffDNA fragments, its methylation status, and the sex of the fetus. Both HMGB1 and cffDNA have also been detected in hAEC-derived exosomes which are upregulated in the cytoplasm in response to OS in vitro [10]. OS has been thought to contribute to FM weakening by inducing an enzymatic breakdown of the extracellular matrix in the amniochorion [15], although it is unclear what the mechanism is that precipitates the induction of OS in these tissues. A clear understanding of how cellular stress, such as OS, is induced in the FM may provide insight into how DAMPs, such as HMGB1 and cffDNA, are released in the amniotic cavity where they can contribute to FM inflammation and subsequent weakening in utero.

Cellular stressors, such as stretch and OS, may significantly contribute to FM weakening by inducing a proinflammatory response that induces extracellular matrix (ECM) degradation in the FM that precedes its rupture [9,16,17,18]. Overlapping cellular stress signaling pathways have been shown to be important for these processes, including those mediated by proinflammatory Nuclear factor kappa B (NF-κB) activation—a transcription factor that is important for the timing of human parturition [9]. Our recent findings demonstrate that stretch of hAECs induces NF-κB activation, reactive oxygen species (ROS) production, Nuclear factor erythroid 2-related factor 2 (Nrf2) downregulation, and the release of the DAMP HMGB1 [9]. This work also showed that concurrent treatment with the phytochemical sulforaphane (SFN) (2 μM) prevented the stretch-induced decrease in Nrf2 expression which is normally associated with labor and preterm premature rupture of FMs [19,20]. SFN is well studied for its potent inhibitory effects on inflammation and may reduce cellular stress associated with DAMP signaling in other tissues [21].

As FMs are significantly stretched in vivo, we postulate that stretch is an important trigger for FM weakening that precedes the induction of inflammation and OS. DAMPs are released by FMs in response to OS and inflammation, and therefore they may be the key link between stretch and the initiation of FM weakening. Thus, the goal of this work was to examine the response of human FMs to both HMGB1 and cffDNA and to determine whether stretch is also a stimulus for cffDNA release. We aimed to achieve the following: (1) ascertain whether stretched hAECs secrete cffDNA, (2) determine if fetal sex influences OS and HMGB1 release in explants treated with cffDNA, and (3) test if SFN treatment can modulate inflammatory signals (cytokine and DAMP secretion) from stretched hAECs [9]. A thorough understanding of DAMP signaling in FMs may aid in our understanding of how these molecules can contribute to the processes of FM weakening during human parturition.

## 2. Results

### 2.1. cffDNA Is Secreted by Stretch and It Increases Extracellular ROS and HMGB1 Secretion

**Figure 1:**
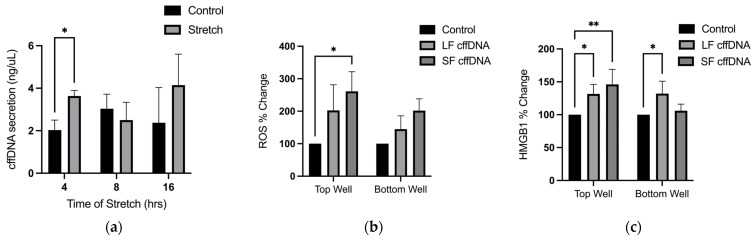
Stress response of cffDNA treated FM tissue. (**a**) Graph of quantified cffDNA secretion (*n* = 4) over 4, 8, and 16 h of stretch and control. (**b**) HMGB1 secretion in top and bottom compartments with control, LF cffDNA, and SF cffDNA. (**c**) ROS quantification of top and bottom compartments. Data displayed as mean ± SEM. * *p* < 0.05, ** *p* < 0.01 two-way ANOVA followed by Dunnett’s multiple comparison test.

Stretch has been implicated as one of the primary triggers for FM rupture through its capacity to induce cellular stress and inflammation. Therefore, as we have already shown that cffDNA can induce cytokine and MMP production [13], we sought to further assess its role in FM weakening by determining whether stretch can cause its release from hAECs. Cells were subjected to 20% cyclic stretch for 4, 8, and 16 h (Figure 1a) and cffDNA secretion was determined by spectrophotometry. After 4 h of stretch, cffDNA secretion in the conditioned media had significantly increased by 78.7% (*n* = 3, *p* < 0.05) in comparison to the no-stretch control. As we have previously demonstrated that stretch induces other DAMP secretion (HMGB1) and ROS production in hAECs, we next determined if cffDNA would induce their production, akin to a cellular stress response independent of cellular stretch. Human FM explants held in Transwell holders were treated with 100 ng/mL of either large fragment cffDNA (LF cffDNA > 1000 bp) or small fragment (SF cffDNA < 200 bp) cffDNA to the apical side of the explant (as previously described [13]). After 24 h, ROS and HMGB1 were quantified in conditioned and non-treated control explant supernatants (*n* = 6) collected from both apical (amnion/fetal) and basal (decidua/maternal) compartments. Apical ROS generation, as determined by the production of DCF (Figure 1b), was significantly increased by 161% (*p* < 0.05) with SF cffDNA treatment in comparison to the controls. There were no significant changes in ROS generation with LF cffDNA in the apical compartment. In the basal compartment, there were no significant changes with either LF or SF cffDNA. Next, the secretion of HMGB1 was measured by ELISA assay (Figure 1c). Apical secretion of HMGB1 was significantly increased by 31.59% (*p* < 0.05) and 46.13% (*p* < 0.01) with LF cffDNA and SF cffDNA, respectively. In the basal compartment media, HMGB1 secretion was increased with LF cffDNA by 32.05% (*p* < 0.01). There were no significant differences in HMGB1 secretion with SF cffDNA secretion in the basal compartment media.

### 2.2. The Induction of ROS and HMGB1 Secretion from FM Explants Are Dependent on the Fetal Sex of the cffDNA

**Figure 2:**
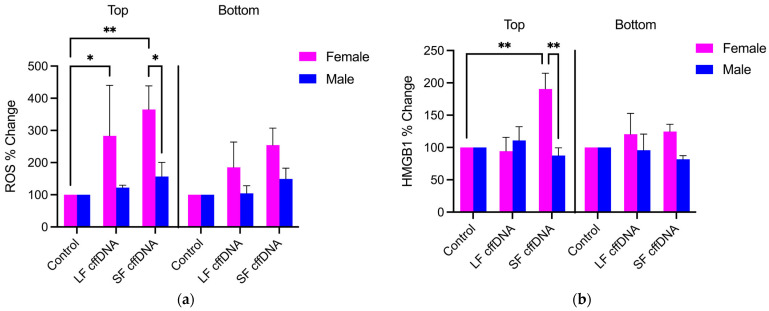
Sex-specific effects of cffDNA treatment. (**a**) ROS quantification of top and bottom compartments by gender and DNA treatment. (**b**) Top and bottom compartment of HMGB1 secretion fold change of DNA treatment over controls. Data displayed as mean ± SEM. * *p* < 0.05, ** *p* < 0.01 two-way ANOVA followed by Tukey’s multiple comparison test.

As we showed that cffDNA increases ROS and HMGB1 (Figure 1b,c) and have previously shown that the FM cytokine secretion response to cffDNA is fetal sex-specific [13], we next ascertained whether the production and secretion of ROS and HMGB1 are also dependent on fetal sex. The data were analyzed by identifying male and female stress responses (*n* = 6) (Figure 2) to LF cffDNA and SF cffDNA to uncover any sex-specific patterns in ROS production (Figure 2a) and HMGB1 secretion (Figure 2b). This analysis demonstrated that the males did not produce significant changes in ROS generation or HMGB1 release in response to either LF cffDNA or SF cffDNA. In contrast, the female fetuses demonstrated significant changes: 183.1% (*p* < 0.05) and 265.3% (*p* < 0.01) increases in apical ROS production (Figure 2a) in response to LF cffDNA and SF cffDNA, respectively. Female fetus HMGB1 release (Figure 2b) significantly increased by 90.59% (*p* < 0.01) in the apical compartment only. When comparing the differences between sexes, females had a significantly greater (*p* < 0.05) apical response to SF cffDNA in the apical compartment media in comparison to the male fetuses (Figure 2a). Both males and females did not have any observable responses in the basal compartment media (Figure 2a,b).

### 2.3. The Receptors RAGE, TLR-9, and Signaling Molecule STING Are Expressed in Cells throughout the Layers of the FM

**Figure 3:**
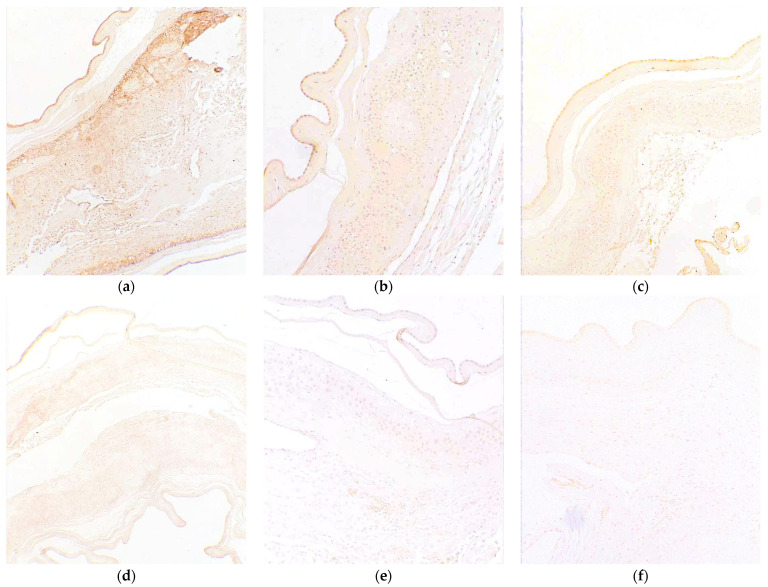
Target receptor expression (top row) in amnion, chorion, and decidua cells: (**a**) RAGE (ab3611, 1:100), (**b**) STING (ab189430, 1:25), and (**c**) TLR-9 (ab37154, 1:25). Immunoglobulin negative control (bottom row): (**d**) RAGE IgG, (**e**) STING IgG, and (**f**) TLR-9 IgG. (*n* = 9) Magnification 100×.

As we have shown that cffDNA is able to increase ROS, HMGB1 (Figure 1 and Figure 2), and various proinflammatory cytokines [13], we next sought to confirm the expression of FMs’ known receptors, the receptor for advanced glycation endproducts (RAGE) and Toll-like receptor 9 (TLR-9), and an important signaling molecule known as stimulator of interferon genes (STING) [22] from full thickness FMs (Figure 3). Our data illustrating that the cffDNA response characteristics are dependent on fetal sex (Figure 2), size, and methylations status have led us to assume that these cffDNA species may operate through differential binding to its known receptors or signaling, which activate various NF-κB pathways [22,23,24,25]. Using immunohistochemistry (IHC) and a qualitative scoring measure (Immunoreactive Score), the relative expression of these proteins was calculated based on the intensity of the staining and the percentage of cells stained in each cell in the FM. IHC analysis of FMs (*n* = 9) confirmed the expression of all three proteins throughout the amnion, chorion, and decidua (Figure 3a–c) at a moderate to strongly positive level for all receptors in all samples of FMs. The RAGE (Figure 3a) receptor had the strongest overall signal. RAGE and TLR-9 expression were highest in hAECs (Figure 3a,c), whereas STING (Figure 3b) expression was mostly expressed in hAECs and chorion cells. HMGB1 is able to initiate a proinflammatory response in the FM by activation of RAGE and TLR-9 after complexing with cffDNA or working independently of cffDNA [26].

### 2.4. HMGB1 Causes the Translocation of NF-κB in hAECs

**Figure 4:**
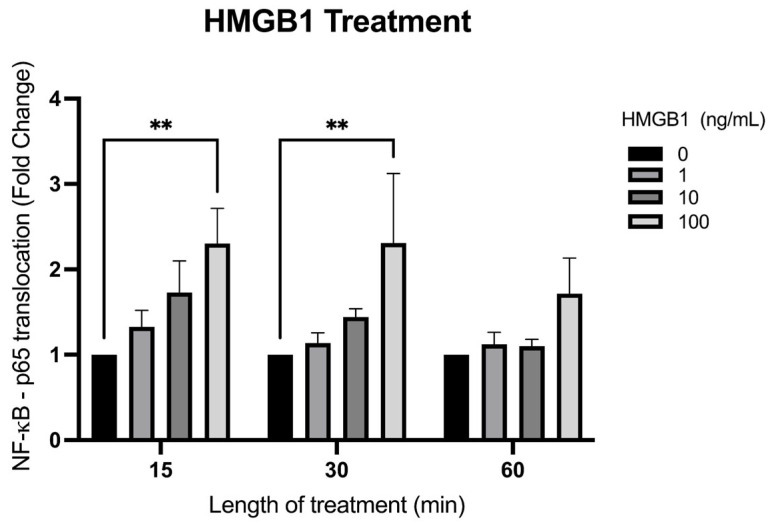
Time-course treatment quantification of p65 NF-κB translocation with 0, 1, 10, and 100 ng/µL of HMGB1. Data displayed as mean ± SEM. (*n* = 3) ** *p* < 0.01 two-way ANOVA followed by Dunnett’s multiple comparison test.

In order to further characterize its proinflammatory response in the FM, we first treated hAECs with increasing concentrations of HMGB1 (0, 1, 10, 100 ng/μL) and quantified nuclear translocation of NF-κB subunit p65 after 15, 30, and 60 min of treatment to confirm this response. In comparison to the non-treated controls, NF-κB subunit p65 translocation was significantly increased (*n* = 3, *p* < 0.01) at the 15 and 30 min time point with 100 ng/μL of HMGB1 (Figure 4).

### 2.5. Proinflammatory Cytokine Gene Expression in Response to HMGB1 Is Amnion Cell-Type Specific

**Figure 5:**
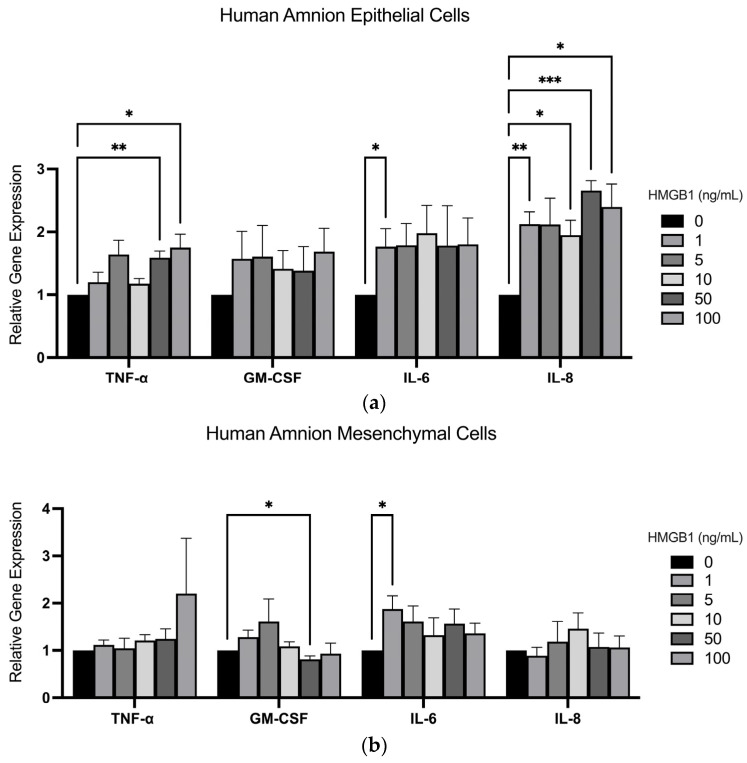
Expression of cytokines in response to HMGB1 in amnion cells. Relative expression of proinflammatory cytokines TNF-α, GM-CSF, IL-6, and IL-8 RNA in (**a**) hAECs and (**b**) hAMCs (normalized to GAPDH and 18S) in response to HMGB1 treatment. Data displayed as mean ± SEM. * *p* < 0.05, ** *p* < 0.01, *** *p* < 0.001 one-way ANOVA followed by multiple *t*-test.

Previous studies have suggested that intraamniotic HMGB1 can cause the production of proinflammatory cytokines from the chorioamniotic membranes mediated by RAGE and TLR-9 [14,27,28]. Thus, we confirmed their expression (Figure 3) in the FMs and the ability of HMGB1 to cause the activation of the proinflammatory transcription factor NF-κB in this tissue (Figure 4). The gene expression of proinflammatory cytokines (TNF-α, GM-CSF, IL-6, and IL-8) were then measured and the responses seen were different from hAECs (Figure 5a) and human amnion mesenchymal cells (hAMCs) (Figure 5b) after 2 h of treatment with HMGB1 (1, 5, 10, 50, and 100 ng/mL). HMGB1 significantly increased the expression of cytokines TNF-α, IL-6, and IL-8 in hAECs (Figure 5a). However, in hAECs, TNF-α (*n* = 5) was significantly increased by 58.93% (*p* < 0.01) and 75.06% (*p* < 0.05) with 50 and 100 ng/mL HMGB1, respectively. hAEC expression of IL-6 (*n* = 8) was significantly increased by 76.4% (*p* < 0.01) by the lowest concentration of HMGB1 (1 ng/mL). IL-8 expression in hAECs (*n* = 5) was increased by 102.4% (*p* < 0.01), 94.73% (*p* < 0.05), 165.6% (*p* < 0.001), and 139.6% (*p* < 0.05) with 1, 10, 50, and 100 ng/mL HMGB1, respectively. There were no significant changes in GM-CSF expression with HMGB1 treatment in hAECs (*n* = 8). However, in hAMCs (*n* = 6) (Figure 5b), there was a significant decrease in GM-CSF expression by 18.7% (*p* < 0.05) with 50 ng/mL HMGB1 and a significant increase in IL-6 expression by 87.29% (*p* < 0.05) with 1 ng/mL HMGB1. hAMC expressions of TNF-α and IL-8 did not significantly change in response to HMGB1.

### 2.6. Sulforaphane Inhibits Secretion of DAMPs and Modulates Cytokine Release in hAECs

**Figure 6:**
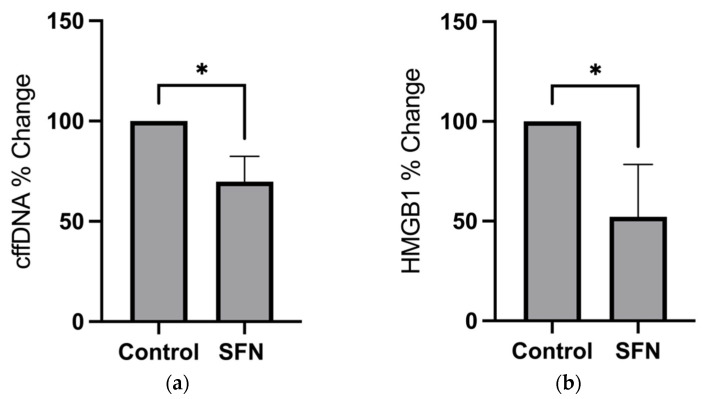
(**a**) cffDNA and (**b**) HMGB1 secretion in stretched and non-stretched (control) hAECs treated with or without 2 µM of SFN. Nanodrop and ELISA assay quantification methods were used to measure secretion of cffDNA (*n* = 4) and HMGB1 (*n* = 3), respectively. Data displayed as mean ± SEM. * *p* < 0.05 paired *t*-test.

Our work has collectively demonstrated that stretching isolated hAECs elicits the secretion of HMGB1 and cffDNA and that both of these DAMPs can lead to cytokine release from FMs and also individual cells of the amnion. As SFN treatment has been shown to activate transcription factor Nrf2, a key regulator of the cellular compensation response to stress [9], we ascertained whether SFN would inhibit stretch-induced DAMP release in hAECs (Figure 6). A total of 2 μM SFN significantly decreased cffDNA release in stretched hAECs (*n* = 4) by 24.25% (*p* < 0.05). HMGB1 secretion was significantly decreased by 27.89% (*p* < 0.05) in control hAECs (Figure 6b). In stretched hAECs, HMGB1 secretion was significantly decreased by 29.69% (*p* < 0.05) and 29.34% (*p* < 0.05) with 2 μM SFN. 

**Figure 7:**
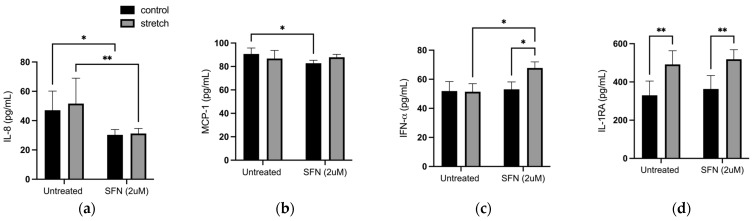
Luminex quantification of secreted cytokines (**a**) IL-8, (**b**) MCP-1, (**c**) IFN-α, and (**d**) IL1-RA. Data displayed as mean ± SEM. (*n* = 5) * *p* < 0.05, ** *p* < 0.01 two-way ANOVA with Fisher’s LSD test.

As SFN treatment is known to downregulate inflammation as a potent protective phytochemical [29], we then measured its effect on the secretion of 24 cytokines after stretching, and control hAECs (Figure 7), treated with 2 μM SFN. Of the cytokines detected by this assay, secretion of interferon alpha (IFN-α), interleukin-1 receptor antagonist (IL1-RA), monocyte chemoattractant protein-1 (MCP-1), and IL-8 were significantly altered with stretch, SFN, or a combination of both. IL-8 secretion was significantly decreased with SFN by 39.5% (*p* < 0.01) and 55.28% (*p* < 0.01) in stretched and control hAECs, respectively (Figure 7a). MCP-1 secretion was slightly decreased by 9.57% (*p* < 0.05) with SFN in stretched cells (Figure 7b). Secretion of the antiviral cytokine IFN-α (Figure 7c) was increased with SFN (*p* < 0.05) only in stretched hAECs. Stretch alone (no SFN treatment) did not increase IFN-α secretion. The IL1-RA was not affected by SFN treatment but was significantly increased with stretch by 104.3% (*p* < 0.01) in comparison to control (untreated) hAECs (Figure 7d).

## 3. Discussion

**Figure 8:**
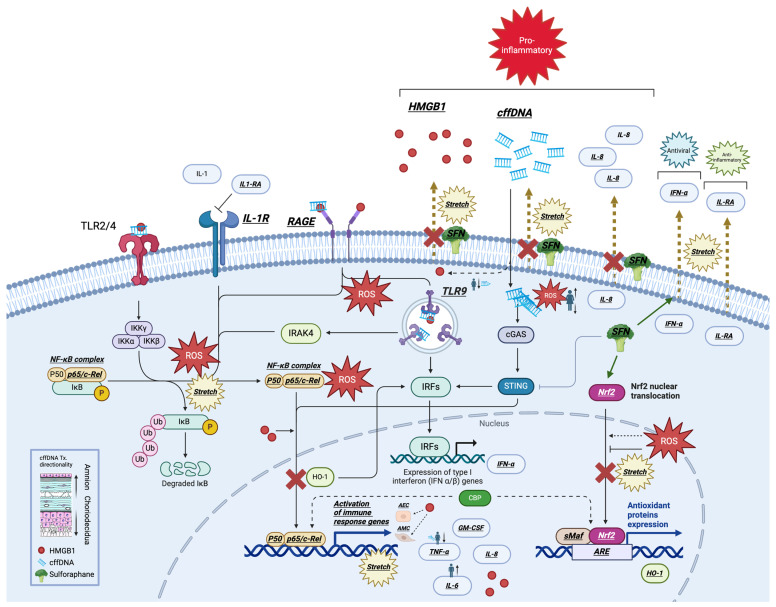
Cellular stress pathways as a result of stretch and DAMP signaling. hAEC stretch induces release of HMGB1 and cffDNA. TLR-2/4/9 and RAGE receptors may be activated by HMGB1 and cffDNA. IL1-RA is secreted as a regulatory mechanism of stretch for the inhibition of IL-1 receptor. Activation of these receptors lead to the induction of NF-κB or IRFs that initiate expression of cytokines. SFN can inhibit the cellular stress response to stretching.

The goal of this study was to increase our understanding of the role of stretch in the initiation of parturition through its downstream production of DAMPs, building upon our previous work focused on these key mediators of pregnancy. Our new data add to the increasing body of evidence that support the role of cellular stressors (such as stretch) as key contributors to FM weakening through the initiation of a proinflammatory signature [5,9]. Taken together, our data suggest that stretch can induce cellular stress and inflammation through the DAMPs HMGB1 and cffDNA by autocrine signaling in the amnion (Figure 8). To our knowledge, there are no clinically approved anti-inflammatory medications that are effectively used to prevent or delay rupture of membranes or chorioamnionitis. Thus, part of the ultimate goal of this work is to increase our knowledge of these processes and to explore the potential for anti-inflammatory therapeutics in clinical practice.

We have previously demonstrated that the in vitro stretch of hAECs reduces Nrf2 expression, induces HMGB1 secretion [9], ROS generation, the activation of NF-κB, and the subsequent secretion of several proinflammatory cytokines. However, the findings of this study now show that the DAMP cffDNA is also secreted after stretching cells from the amnion (Figure 1a). Like HMGB1, cffDNA is also found in the intraamniotic cavity and may consequently contribute to FM rupture by adding to the inflammatory load associated with the onset of parturition (Figure 8. After 4 h of stretch, cffDNA release was significantly increased in hAECs in comparison to unstretched controls (Figure 1a); after 8 h and 16 h of stretch, there were no significant changes in cffDNA secretion. We believe this is due to the inherent cellular stress that results from the extended time that the cells remain in a serum-starved culture medium in this model system. Our treatment of FM explants with small and large fragments of cffDNA illustrated that SF cffDNA induced a stronger stress response as demonstrated by greater increases in ROS (Figure 1b) and HMGB1 (Figure 1c). This supports our previous data showing that smaller fragments of cffDNA are a more potent stimulator of key proinflammatory cytokines for FM weakening [13].

In addition to cffDNA size, fetal sex has also previously been shown to play a role in maternal cytokine production and may therefore also affect the magnitude or specific players involved in an inflammatory load [30]. Recently, we showed that cffDNA induces the release of MMPs and proinflammatory cytokines in the FM and that the specific response is dependent on the sex of the fetus [13]. Our new data also illustrate the importance of fetal sex as female fetuses’ stress responses of increased ROS generation (Figure 2a) and HMGB1 release (Figure 2b) were more robust than those from male fetuses. As female fetuses are known to be associated with improved pregnancy outcomes [31], the cellular stress markers ROS and HMGB1, which can act as secondary messengers, may be involved in downstream signaling mechanisms associated with reduced inflammation in late pregnancy [31]. Thus, fetal sex-specific differences in the known cffDNA receptors or signaling molecules involved may explain this discrepancy in cellular stress between the sexes [25,32]. Although our apical treatment of FM explants with cffDNA resulted in a significant apical stress response (Figure 2), changes were seen in the basal side too (Figure 2), which we believe would have produced a significant response if given more time to build up in the media. This is interesting because, taken with our previous data showing the propagation of NF-κB activation through the whole thickness of the FM [13], these data add credence to the hypothesis that effectors in the amniotic fluid can have a powerful effect on the whole of the FM and signal to underlying maternal tissues.

The DAMPs produced in the apical compartments of female fetus explants treated with SF cffDNA (Figure 2) were significantly greater than their male counterparts. These smaller cffDNA fragments have an increased capacity to bind and activate the DAMP receptor TLR-9 compared to larger cffDNA fragments [25]. Thus, we confirmed the expression of this receptor throughout the FM (Figure 3c). The hAECs, which line the innermost apical side of the FMs, may function as the primary “receptor” of the entire FM due to its robust expression of receptors [24,33,34,35,36,37]. However, we saw the expression of this receptor throughout the different cells of the FM, and thus the cffDNA may travel through this tissue to activate these receptors too, as it has been identified in exosomes isolated from the amnion [10,38].

In addition to TLR-9, we also demonstrated that all of the layers of the FM robustly express the RAGE receptor (Figure 3a) and STING (Figure 3b) signaling molecule. RAGE can also bind PAMPs, such as LPS and bacterial/viral DNA [26,27,39,40], and DAMPs, such as HMGB1 and cffDNA [25,41,42,43,44,45]. Thus, in the presence of cellular stressors, such as stretch, OS, and infection, hAECs have been shown to induce a proinflammatory stress response marked by NF-κB activation, secretion of proinflammatory cytokines, and secretion of HMGB1 and cffDNA in vitro [11,46,47,48,49]. In addition to cffDNA, the receptors RAGE and TLR-9 have been shown to bind HMGB1 or HMGB1 complexes with cffDNA.

Both cffDNA and HMGB1 are two proinflammatory DAMPs that may contribute to FM weakening by inducing proinflammatory NF-κB activation and the downstream production and secretion of cytokines and MMPs in FMs. Increased proinflammatory NF-κB activity is associated with the onset of labor [50] and is thought to be central to several prolabor pathways (Figure 8). In addition to our previous study focused on cffDNA [13], this study confirms that HMGB1 may also participate in the onset of labor by binding hAEC receptors (Figure 3), resulting in the activation of NF-κB (Figure 4) and subsequent production of proinflammatory cytokines (Figure 5a). The expression of these known DAMP receptors may play a role in the differential effects of cffDNA size, fetal sex, and methylation status on inflammation and cellular stress observed in our work.

This study is the first to identify potential differences in the proinflammatory cytokine expression profiles of hAECs and hAMCs in response to HMGB1 (Figure 5). The varied cytokine expression profiles of hAECs and hAMCs in response to HMGB1 may reflect their differing roles in FM physiology. In contrast to hAMCs, hAECs, which have a high expression of DAMP and PAMP receptors (Figure 3), significantly upregulate TNF-α and IL-8 expression in response to HMGB1 (Figure 5a). These cytokines are thought to increase MMP production and activation in FMs [51]. In addition to its role as a barrier cell, these data suggest that hAECs have an indirect role in ECM turnover and a greater role than hAMCs in the FM inflammatory response to proinflammatory markers found in the amniotic cavity. In contrast to hAECs, hAMCs upregulate MMP-1 and MMP-9 in response to thrombin, suggesting a direct role in ECM turnover [52,53]. Therefore, HMGB1 may contribute to the initiation of labor by inducing chorioamniotic inflammation through hAEC binding, leading to the activation of MMPs produced by hAMCs that contribute to FM weakening and subsequent rupture.

We have previously demonstrated that cffDNA is also able to induce chorioamniotic inflammation and MMP activation [9]. The data in our current study suggest that the cffDNA released by stretch can contribute to HMGB1 release as a potential feedforward mechanism of stretch, indicating stretch as a potent stimulus for DAMP release in utero (Figure 8). Treatment with Nrf2 activator SFN was shown to significantly modify the hAEC response to stretch (Figure 8), including the downregulation of both HMGB1 (Figure 6a) and cffDNA (Figure 6b) in stretched cells. Interestingly, our previous data demonstrated that SFN does not inhibit stretch-induced ROS production in hAECs [9], implying that SFN may inhibit cellular stress and modify cytokine release independently of ROS.

In this study, SFN treatment had a variable effect on the secretion of human cytokines IL-8, MCP-1, and IFN-α in hAECs (Figure 7). IL-8 (Figure 7a) is a cytokine widely accepted to be involved in the initiation of normal term parturition and in infection-induced preterm birth [47,48]. Its secretion is decreased with SFN treatment in both stretched and unstretched control cells. A longer period of stretch may be required in order to observe significant changes in its secretion as we have previously shown that it is significantly upregulated and released with 24 h of stretch in hAECs [47,48]. SFN may therefore inhibit the proinflammatory signaling of hAECs involved in the initiation of parturition by inhibiting IL-8 secretion. We have previously demonstrated that inflammatory IL-8 secretion occurs at much longer timepoints, consistent with the idea that cellular stress occurs acutely following cytokine release, and that DAMPs such as HMGB1 are constitutively expressed, while cytokines are expressed de novo upon the introduction of a stimulus. MCP-1 is a chemokine that induces the expression of the proinflammatory cytokines IL-6 and IL-1β, which in turn may regulate the expression of TGF-β involved in collagen stimulation [54]. MCP-1 is decreased with SFN non-stretched cells (*p* < 0.05) (Figure 7b), indicating that SFN can affect the TGF-β-mediated collagen remodeling of FMs through its regulation of MCP-1. IFN-α is part of the interferon class of cytokines known for their role in the innate immune response involved in the inhibition of viral replication and dissemination [55]. IFN-α secretion (Figure 7c) was only increased (*p* < 0.05) with SFN treatment in cells that were stretched, indicating that SFN treatment may be effective for the enhancement of the fetal antiviral defenses only in cells that are “primed” with stretch. 

SFN did not affect the release of the IL-1 receptor antagonist (IL-1RA), although it was significantly increased by stretch (*p* < 0.01) (Figure 7d). This anti-inflammatory cytokine is known to be released by amnion, chorion, and predominantly decidua, although only decidual cell stimulation with LPS and IL-1 was shown to increase its release in vitro. IL-1β, TNF-α, and IL-6 [56,57,58] are all upregulated in the context of inflammation and infection and are involved in the pathogenesis of preterm labor. It was previously demonstrated that maternal decidua secretes additional IL-RA in the presence of a female conceptus [31], suggesting that females are better protected against pregnancy. The secretion of IL-RA may serve as a regulatory mechanism to control the proinflammatory effects of stretch.

As an inducer of cellular stress, these data support the hypothesis that stretch forces are central to the FM weakening process. The membranes are significantly distended in utero and are then subject to additional pre-labor stretch forces by Braxton Hicks contractions [59,60,61]. In the context of pregnancy pathology, the overdistension of FMs by a multiple pregnancy or polyhydramnios is associated with an increased risk of preterm birth [62]. The primary findings of this study collectively demonstrate that stretch (1) secretes cffDNA (Figure 8), (2) cffDNA produces a sex-specific cellular stress response (Figure 8), and (3) SFN inhibits the cellular stress response to stretch (Figure 8). Indeed, stretch is clearly implicated as an important trigger for the initiation of cellular stress and inflammation in FMs and DAMPs are the primary mediators of this proinflammatory process that precipitates FM weakening and then rupture. To address the important issue of preterm premature rupture of membranes (pPROM), more work is needed to identify the factors that contribute to FM stress and the triggers that initiate the normal FM weakening process such as fetal sex and stretch, respectively. Fetal sexing that is primarily determined using ultrasound imaging, and maternal peripheral blood analysis, could be linked to future studies aimed at ascertaining whether fetal sex is associated with stress markers and inflammatory pathologies in pregnancy that could be measured through clinical measures such as maternal blood analysis, amniocentesis, and related methods. This study identifies the ability of SFN to inhibit the cellular stress effects of stretch that are thought to participate in FM weakening and how certain stress mechanisms are influenced by fetal sex such as the cffDNA-induced HMGB1 release that disproportionately affects females. Continuing to expand our understanding of the mechanisms involved in FM weakening may uncover potential therapeutic modalities that may delay FM rupture, such as DAMP inhibition, and how factors such as fetal sex can impact pregnancy outcomes and effectiveness of therapeutic treatment.

## 4. Materials and Methods

### 4.1. Tissue Collection and Culture of Human Amnion Epithelial Cells

FMs (total of 29) were obtained from term repeat cesarean section live births at Kapiolani Women and Children’s Hospital (Honolulu, HI, USA) with IRB approval. All tissues were examined by a pathologist for histological evidence of infection; if positive, they were excluded from further analysis. The FMs were isolated 1 inch from the placenta and washed in sterile phosphate-buffered saline (PBS) within 30 min of collection to remove blood. hAECs were isolated as previously described [48]. In short, the amnion was peeled away from the chorion and subsequently washed with PBS. The tissue was then minced and incubated three times with 2 g/L trypsin (SAFC; Buchs, Switzerland) in Dulbecco’s modified Eagle medium (DMEM) (Life Technologies Limited; Paisley, UK) at 130 rpm at 37 °C for 30 min. Resultant individual cells were pelleted by centrifugation, and they were suspended in media (F12:DMEM; 1:1 *v*/*v*) (Invitrogen; Carlsbad, CA, USA) with fetal bovine serum (FBS) (10%, *v*:*v*), 1% penicillin (200 U/mL), and 100 µg/mL streptomycin (Life Technologies; Burlington, ON, Canada). 

### 4.2. Cell Culture and Stretch of hAECs 

Cells were seeded at 1 × 106 cells per well on a collagen IV-coated silicone-lined stretch plate (Flexcell Inc., Burlington, NC, USA). After 4–10 days, the cells reached 70–80% confluency and the media were replaced with 0.5% FBS DMEM/F12 for 12–16 h. Fresh 0.5% FBS DMEM:F12 treated with and without (control) 2 μM SFN (MilliporeSigma; Burlington, MA, USA) was added before addition of stretch stimulus. Cyclic stretch and release were performed using the Flexcell-FX5000TM tension system (Flexcell; Burlington, NC, USA). The 20% cyclic stretch and release experiment was performed as previously described [9,47] for 4, 8, and 16 h with an interval of 27 s of 20% stretch, followed by 7 s of release of this stretch to 0%.

### 4.3. ROS Assay

The condition medium from the top and bottom wells of cffDNA treated (SF cffDNA or LF cffDNA) and untreated (control) FM explants were collected. They were centrifuged for 10 min at 10,000× *g* and 4 °C to remove cell debris. ROS was quantified using OxiselectTM ROS Assay Kit (Cell Bio Labs Inc.; San Diego, CA, USA) as per manufacturer’s instructions. ROS content was determined via dichlorofluorescin production in comparison with the predetermined DCF standard curve and normalized to total protein concentrations using protein assay compatible with conditioned media (Pierce, Waltham, MA, USA).

### 4.4. HMGB1 ELISA 

The condition medium from the top and bottom wells of cffDNA treated (SF cffDNA or LF cffDNA) and untreated (control) FM explants, and conditioned media from stretched and unstretched SFN treated cells, were collected and centrifuged for 10 min at 10,000× *g* and 4 °C to remove cell debris. Medium was then analyzed for HMGB1 secretion as per manufacturer’s instructions using the Human HMGB1 ELISA Kit (LifeSpan BioSciences Inc.; Seattle, WA, USA). Concentrations of HMGB1 were normalized to total protein concentrations using protein assay compatible with conditioned media (Pierce, Waltham, MA, USA).

### 4.5. DNA Isolation from Amnion Epithelial Cells, Sonication, and Verification

DNA isolation was performed using the QIAamp DNA Mini isolation kit (Qiagen, Hilden, Germany) from isolated primary AECs following the manufacturer’s instructions. DNA was quantified using a NanoDrop (Thermo Fisher Scientific, Waltham, MA, USA). To create smaller fragments of cffDNA that would be more like those seen in AF, it was sonicated as previously described [13]. Non-sonicated “whole DNA” was designated as LF cffDNA and 4-min sonicated DNA was designated as SF cffDNA. cffDNA fragment size of the LF cffDNA and SF cffDNA samples were verified on a 2% agarose gel with ethidium bromide (Invitrogen, Waltham, MA, USA) and visualized under UV transillumination using a Chemidoc (Bio-Rad, Hercules, CA, USA) as previously described [13].

### 4.6. cffDNA Treatment of FM Explants

The full thickness amnion/choriodecidua membrane integrity was assessed to ensure all layers were intact with no separation of layers. The membranes were rinsed in sterile PBS and then cut into 2.5 cm^2^ × 2.5 cm^2^ pieces and placed onto sterilized Transwell frames (Corning Inc., Corning, NY, USA) without synthetic membrane. The two-compartment system was created by placing an elastic latex dental band around the as previously described [13]. The amnion side of the FM tissue faced apical to the Transwell insert creating the inner, upper well, while the decidua layer faced outward creating a completely separate, outer, lower well as previously described [13]. Each mounted FM was placed in a single well within a 12-well tissue culture plate with DMEM/F12 medium on both sides of the membranes to allow equilibration for 24 h. The culture medium was removed and the apical side (upper well) of the FM compartment was treated with 100 ng/mL SF cffDNA (small fragment cffDNA < 200 bp) or LF cffDNA (large fragment cffDNA > 1000 bp) in 500 µL DMEM/F12 media and the basal side (lower well) was supplemented with only 1.5 mL DMEM/F12 media for 24 h. The condition medium from the top and bottom wells was collected and centrifuged for 10 min at 10,000× *g* and 4 °C to remove cell debris.

### 4.7. DNA Sex Determination

To determine the sex of each cffDNA sample, polymerase chain reaction (PCR) was performed to identify the presence of sex region y gene (SRY) for males or Alanine aminotransferase-1 gene (ALT1) for females. The sequences of primers for SRY were 5′-CATGAACGCATTCATCGTGTGGTC-3′ and 5′-CTGCGGGAAGCAAACTGCAATTCTT-3′, and 5′-CCCTGATGAAGAACTTGTATCTC-3′ and 5′-GAAATTACACACATAGGTGGCACT-3′ for ATL1 [63]. Each PCR reaction consisted of 100 ng DNA, 1X PCR buffer minus Mg, 0.2 mM dNTP mixture, 1.5 nM MgCl_2_, 0.5 μM SRY and ALT1 primers (FWD and REV), and 2.5 units Taq DNA Polymerase (5U/μL) (Invitrogen, Waltham, MA, USA). All PCR reactions were performed in a thermal cycler (PTC-225, Peltier Thermal Cycler, MJ Research, Manchester, NH, USA) at 94 °C (3 min) for initial DNA denaturation, followed by 35 cycles of 94 °C (15 s) for DNA denaturation, 55 °C (30 s) for primer annealing, and 72 °C (90 s) for primer extension, with a final extension of the cycle at 72 °C (10 min). The amplified PCR products were separated on a 2.5% agarose gel with ethidium bromide and imaged under UV transillumination using a Chemidoc (Bio-Rad, Hercules, CA, USA). The product size of SRY was 254 bp for Y chromosome and ALT1 was 300 bp for X chromosome.

### 4.8. Immunohistochemistry for STING, TLR-9, and RAGE

IHC was performed on paraffin sections (5 mm) using the Vectastain ABC kit (Vector Laboratories; Burlingham, CA, USA) as previously described [9]. In short, FM tissue sections were deparaffinized and hydrated through a series of decreasing alcohol (95%, 80%, 70%, and 60% EtOH; >80 °C, 10 mM citric acid buffer, pH 6.0) for 20 min. Endogenous peroxidase activity was blocked using 0.3% hydrogen peroxide in methanol for 30 min. This was followed by Polyclonal Anti-TLR-9 (ab37154, 1:25), Anti-STING (ab189430, 1:25), and anti-RAGE (Ab3611, 1:100) antibody incubation in 1% bovine serum albumin (EMD Chemicals Inc.; Gibbstown, NJ, USA) in PBS for 1 h at RT. Adjacent tissue served as 1:100 IgG control (R&D Systems; Minneapolis, MN, USA). TLR-9, STING, RAGE, and IgG stained tissues were incubated with a biotinylated secondary antibody for 30 min, rinsed with PBS, and treated with the avidin-biotin-peroxide complex (ABC) (Vector Laboratories, Burlingame, CA, USA) for an additional 30 min. Tissue sections were counterstained with hematoxylin and re-dehydrated. Slides were mounted with Permount (Fisher Chemicals; Pittsburg, PA, USA). Images were taken using the Nikon C1 Plus Ti Eclipse imaging system (Nikon Instruments; Melville, NY, USA).

### 4.9. Immunocytochemistry

Primary isolated AECs (100,000 cells/well) were seeded into 4-well chamber slides and grown to 80% confluency. The AECs were treated with 0, 1, 10, 100, and ng/mL HMGB1. After treatment, the AECs were fixed with 4% paraformaldehyde (PFA) (Sigma Aldrich, St. Louis, MO, USA) in 1X PBS for 15 min, followed by two washes of 1X PBS. Non-specific binding was blocked with 5% bovine serum albumin (BSA) in PBS for 1 h and subsequent incubation with 1:500 rabbit polyclonal anti-NF-κB p65 antibody (06-418, Sigma Aldrich, St. Louis, MO, USA) incubation in 1% BSA in PBS for 1 h at room temperature. Secondary antibody Alexa fluor-488 anti-rabbit at 1:2000 (Life Technologies, Waltham, MA, USA) incubation was performed for 1 h at room temperature. The cells were then washed with PBS and counterstained with DAPI at 1:5000 (Calbiochem, Billerica, MA, USA) for 5 min. The slides were mounted with ClearMount with Tris buffer (Electron Microscopy Sciences, Hatfield, PA, USA) before imaging with confocal microscopy (Nikon C1 Plus Ti Eclipse epi-fluorescence; Nikon Corporation, Tokyo, Japan). The effects of treatment with HMGB1 and LPS lasted for 30 min, were measured as fold change of p65 translocation, and were quantified using immunofluorescent imaging. Cells with observable p65 nuclear localization were determined as p65-positive cells and were measured as a percentage out of the total number of cells observed in a single field of view after HMGB1 and LPS treatment in comparison to non-treated controls. 

### 4.10. RNA Isolation and Quantitative Real-Time PCR

hAMCs and hAECs were isolated from FMs, placed onto a 10 cm^2^ culture plate, and grown to 80% confluency. Cell media were then replaced with 0.5% FBS DMEM/F12 for 12–16 h. They were then treated with HMGB1 (1, 5, 10, 50, and 100 ng/mL) for 2 h. RNA was isolated using RNeasy Mini Kit (Qiagen; Valencia, CA, USA), cDNA was generated with the High Capacity RNA to cDNA kit (Applied Biosystems), and RT-PCR was performed using Taqman Gene Expression assays for Human IL-6, IL-8, TNF-α, and GM-CSF (Thermo Fisher Scientific, Waltham, MA, USA). RNA quality and concentration were quantified using the NanoDropTM Lite Spectrophotometer (Thermo Fisher Scientific; Wilmington, DE, USA). Reverse transcription was performed to convert 0.5 μg of RNA to cDNA using the High-Capacity RNA-to-cDNATM Kit (Thermo Fisher Scientific Baltics UAB; Vilnius, Lithuania). Each 96-well plate included a water blank and a reverse transcriptase blank. Real-time PCR was carried out on an Applied Biosystems StepOne Real-Time PCR System (ABI; Foster City, CA, USA). Each reaction was conducted in triplicate and the results were normalized to the expression of GAPDH and 18S (Hs99999901_s1, Hs02758991_g1, ThermoFisher Scientific, Waltham, MA, USA) in each sample.

### 4.11. Luminex Quantification of Released Inflammatory Signaling Molecules

Conditioned media samples were obtained from SFN treated and not treated hAECs subjected to 4 h stretch and control conditions. Aliquots were immediately centrifuged in 1.5 mL microcentrifuge tubes. Supernatant was collected into a new 1.5 mL microcentrifuge tube and stored at −80 °C. The Luminex Performance Human Immunotherapy Discovery 24-plex (LKTM010, R&D Systems, Minneapolis, MN, USA) was used to analyze protein levels for inflammatory-associated biomarkers (CD40 Ligand, GM-CSF, Granzyme B, IFN-α, IFN-γ, IL-1α, IL-1β, IL-1RA, IL-2, IL-4, IL-6, IL-8 IL-10, IL-12 p70, IL-13, IL-15, IL-17A, IL-33, IP-10, MCP-1, MIP-1α, MIP-1β, PD-L1/B7-H1, and TNF-α). Duplicate assays were performed using 50 μL of 1:1 diluted sample according to manufacturer’s instructions. Fluorescence was measured using the Luminex 200 platform (Luminex Corporation, Austin, TX, USA). Data were collected and analyzed with the Luminex xPONENT 3.1 software (Luminex Corporation).

### 4.12. Statistical Analysis

All statistical analysis was performed using GraphPad Prism 10.1.1 (GraphPad Software, San Diego, CA, USA). The data throughout the figures are expressed as ± standard error of the mean (SEM). All statistical comparisons between the groups were identified by using one-way ANOVA or two-way ANOVA analysis followed by Dunnett’s multiple comparison tests, Tukey’s multiple comparison tests, Fisher’s LSD tests, or by paired *t*-tests, as appropriate. The differences * *p* < 0.05, ** *p* < 0.01, *** *p* < 0.001 were considered statistically significant.

## Data Availability

The data generated during the study are available upon request.
